# Tree species composition mapping with dimension reduction and post-classification using very high-resolution hyperspectral imaging

**DOI:** 10.1038/s41598-022-25404-x

**Published:** 2022-12-03

**Authors:** Szilárd Balázs Likó, László Bekő, Péter Burai, Imre J. Holb, Szilárd Szabó

**Affiliations:** 1 Department of Physical Geography, Faculty of Science, Institute of Geography and Earth Sciences, Eötvös Loránd University, Pázmány Péter Sétány 1/A, Budapest, 1117 Hungary; 2 Remote Sensing Centre, University of Debrecen, Böszörményi út 138., Debrecen, 4032 Hungary; 3 Institute of Horticulture, University of Debrecen, Böszörményi u. 138., Debrecen, 4032 Hungary; 4 Eötvös Loránd Research Network (ELKH), Centre for Agricultural Research, Plant Protection Institute, Herman Ottó út 15, Budapest, 1022 Hungary; 5 Department of Physical Geography and Geoinformatics, Faculty of Science and Technology, University of Debrecen, Egyetem Tér 1., Debrecen, 4032 Hungary

**Keywords:** Forest ecology, Forestry, Wetlands ecology

## Abstract

Tree species’ composition of forests is essential in forest management and nature conservation. We aimed to identify the tree species structure of a floodplain forest area using a hyperspectral image. We proposed an efficient novel strategy including the testing of three dimension reduction (DR) methods: Principal Component Analysis, Minimum Noise Fraction (MNF) and Indipendent Component Analysis with five machine learning (ML) algorithms (Maximum Likelihood Classifier, Support Vector Classification, Support Vector Machine, Random Forest and Artificial Neural Network) to find the most accurate outcome; altogether 300 models were calculated. Post-classification was applied by combining the multiresolution segmentation and filtering. MNF was the most efficient DR technique, and at least 7 components were needed to gain an overall accuracy (OA) of > 75%. Forty-five models had > 80% OAs; MNF was 43, and the Maximum Likelihood was 19 times among these models. Best classification belonged to MNF with 10 components and Maximum Likelihood classifier with the OA of 83.3%. Post-classification increased the OA to 86.1%. We quantified the differences among the possible DR and ML methods, and found that even > 10% worse model can be found using popular standard procedures related to the best results. Our workflow calls the attention of careful model selection to gain accurate maps.

## Introduction

Revealing tree species of a forest is important for sustainable forest management, forest biodiversity, forest ecosystem security^1^. The species composition of forest trees can be important from several aspects, such as quantitative estimation of wood raw material, biomass estimation^1–3^, nature conservation^4^, and invasive tree species^5,6^. Furthermore, the change of the species structure and spatial distribution of forests can be a proxy of climate change monitoring, this aspect makes forests one of the important research areas of the twenty-first century^6,7^.

Basic method of tree species mapping is based on field observations (i.e. recognizing each tree); however, it is time consuming and large areas or regions of impervious dense vegetation cannot be surveyed. As an alternative, remote sensing provides data with different details from small scale (1000 m) to large scale (0.1 m) the from simple identification of forests to species level mapping^8^. It corresponds the needs of modern forestry and functional forest management, requireing detailed information about the trees in digital format, thus, remote sensing can provide valuable information about the species and the structure^9^. Several studies proved that satellite and aerial images are efficient tools to map tree species^5,8,10,11^. However, images of visible bands (red, green and blue, RGB, i.e. traditional color orthopohotos) are not appropriate data for semi-automatic even for forest classification, but having at least a near infra-red spectral band enhances the possbilities (it helps to discriminate the vegetetaion from other green surfaces). While multispectral sensors collect the data in 4–8 spectral bands, hyperspectral sensors measure the radiance in tens or hundreds of narrow spectral bands, which increases the possibility to have detailed, in special cases unique, spectral profiles, which helps to distinguish materials in the process of image classification^12^. In forest applications, hyperspectral imagery is considered a better data source than multispectral images ensuring more discriminated species with high accuracies^8,13^.

Hyperspectral sensors can be mounted in different platforms, usually on aircrafts, but also on satellites and uncrewed aerial systems (UASs). Spatial resolution (ground sampling distance, GSD) also can be an important factor when choosing the proper platform: sensors of UASs can have a GSD of 0.1–0.5 m depending on flight heights; in case of aircrafts it is ~ 1–2 m, and, currently there is one hyperspectral satellite, the PRISMA^14^, with 30 m GSD. The appropriate pixel size for tree species mapping is ~ 1 m^15^.

Although the large number of bands provides detailed spectral profiles for surface objects, we face the issues of correlating bands, i.e. redundant information and overfitting in hyperspectral imagery^16,17^. Thus, finding the predictors (i.e. bands) having the best performance distinguishing tree species is crucial. Possible solutions are the variable selection (feature selection) or the dimension reduction (DR, feature extraction or in statistical term, ordination); both can be efficient and, in this study, we focused on DR using three different techniques with various efficacy^18–22^. DR techniques were also thoroughly studied in image processing, but a comparative evaluation on species level (i.e. with classes having similar spectral profiles) is missing. Accordingly, we involved these three DR methods using hyperspectral data.

DR is a common technique and, especially in hyperspectral image analysis, an important approach to work with fewer variables keeping the explained variance as high as possible, becuse it helps to avoid manage the bands of lesser information and the overfit. We aimed to quantify the efficiency of different DR techniques in image classification with comparing the accuracy metrics. We had the following research questions: (i) which classification algorithm, (ii) which DR method, and (iii) how many components of the ordinations are needed to achieve the highest overall accuracy. Furthermore, we investigated a post-classification segmentation method with object-based reclassification to improve the classification results, and also to overcome the challenge of output maps showing a mix of different species for single trees. We combined the pixel-based approach with the OBIA. Our hypothesis was that post-classification can improve the accuracy and provide clarified maps through easier perception.

## Results

### Comparison of ordinations: overall accuracies

**Figure 1:**
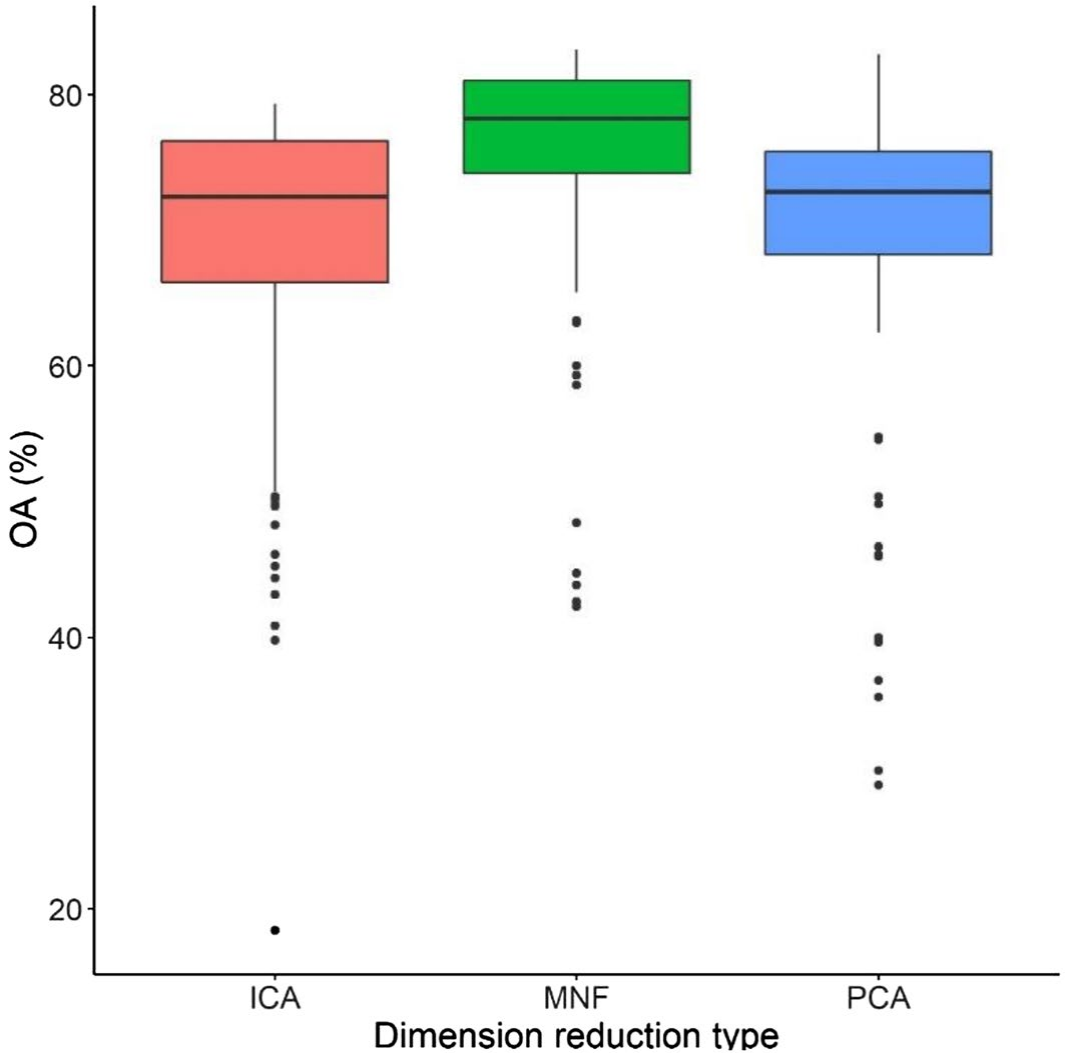
Model accuracies of different dimension reduction techniques. *ICA* Independent Component Analysis, *PCA* Principal Component Analysis, *MNF* Minimum Noise Fraction, *OA* overall accuracy.

Generally, MNF tranformation provided the best OAs (Fig. 1). According to the ANOVA test, differences were significant (F = 17.5, df1 = 2, df2 = 192, *p* < 0.001). Pairwise comparison revealed that MNF provided significantly higher OAs than ICA and PCA (mean diff: − 7.26 and − 7.03, respectively; *p* < 0.05), while PCA components ensured only 0.233 better OAs than ICA which was not significant (*p* = 0.989).

### Number of components and overall accuracies

**Figure 2:**
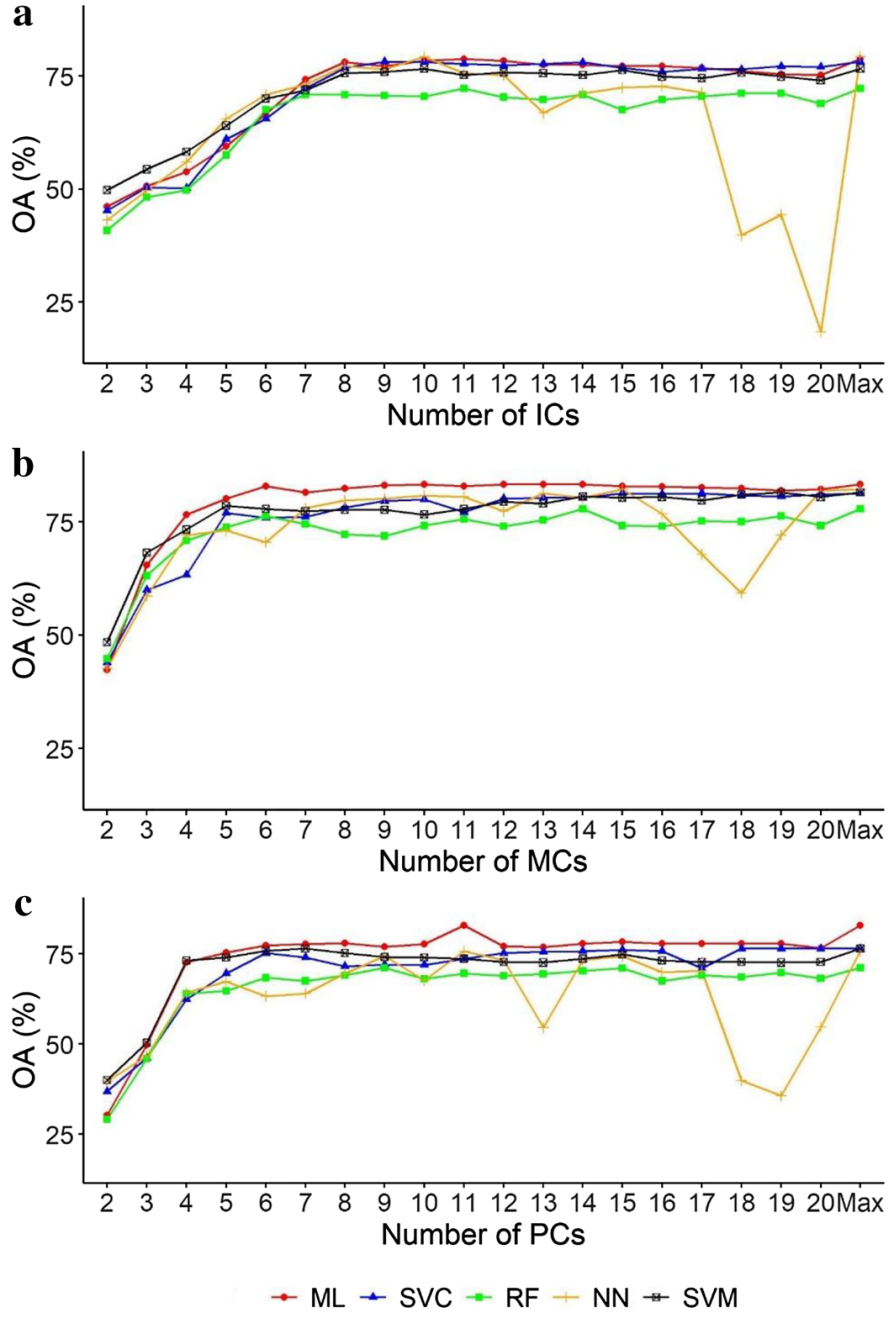
Overall accuracies (OA) and components of Minimum Noise Fraction (**a**), Independent Component Analysis (**b**) and Principal Component Analysis (**c**) by classification algorithms. *MCs* MNF components, *ICs* ICA components, *PCs* PCA principal components, *Alg* algorithms, *ML* Maximum Likelihood, *SVM* Support Vector Machine, *SVC* Linear type of SVM, *RF* Random Forest, *NN* Neural Network.

In case of ICA the Neural Network (NN) provided the best accuracy (79.3%) but it was 4% worse than it was possible to get with the MCs, and it was only the 50th from the total 300 models. Besides, the (Maximum Likelihood) ML classifier with the ICs had 78.8% OA, which was 0.5% worse than of NN. Ideal number of ICs were above 7 to get at least 75% OAs. Usually, Random Forest (RF) was the worse and the ML the best classifier, while the NN’s judgement was ambiguous as the best and the worst OA belonged to it, too (Fig. 2a), therefore, it has the risk that in other cases provides very good or unusable accuracy.

Best accuracies belonged to the MNF transformation with 83.3% OAs (and it was gained with 10, 12, 13, 14 and 21 components, Fig. 2b), and the first 9 models, from the total of 300, were conducted with MNF components and the ML classifier. Even the 9th model of the accuracy rank had an OA of 82.98, even just 0.32% worse than the best one. Model performance improved with higher number of components: from 42% (2 components) to 83.3%, and 7–8 components were the minimum to get better (> 75%) accuracies. The ML had the best performance and considering larger number of components (> 10) generally the RF, in absolute term the NN was the worst (59%) model.

PCA provided better model performance, the maximum OA was gained by the ML classifier (82.98%) with 11 bands (Fig. 2c). In this case, the NN had the worst OAs and the RF was slightly better. The NN, similarly to the experiment with the ICs, had varying performance with worse values, but the best was 7% below the ML’s best model.

### Multivariate statistical evaluation of the overall accuracies

GLM provided a significant model, with an adjusted R^2^ of 0.568. All factors were significant (*p* < 0.001), but from interactions, only the one with the classification algorithms and the number of components was significant (Table 1). According to the effect sizes, largest effect belonged to the number of components (0.468), only the one third was the effect for the transformation type and the classification algorithm (0.169 and 0.148, respectively). Interacdotion of classification algorithms and the number of components had the effect size of 0.08, the half of transformation type’s effect.

**Table 1:** Summary of GLM performed with overall accuracies erosion as target variable.

Source of variation	SS	df	F	*p*	ω^2^*p*
Model	24,565	59	7.663	** < 0.001**	0.567
Ord	3396	2	30.867	** < 0.001**	0.169
Alg	2647	4	12.032	** < 0.001**	0.144
NC	14,644	3	88.745	** < 0.001**	0.468
Ord × Alg	323	8	0.734	0.661	− 0.005
Ord × NC	372	6	1.127	0.347	0.002
Alg × NC	2317	12	3.511	** < 0.001**	0.082
Ord × Alg × NC	866	24	0.656	0.891	− 0.028
Residuals	13,201	240			
Total	1.48e+6	300			

*Ord* dimension reduction type, *Alg* classification algorithms, *NC* number of components, *SS* Sum of Squares, *df* degree of freedom, *F* F-statistic, *p* significance, *ω*^*2*^*p* effect size; × : statistical interactions.

*p* < 0.001 is highlighted with bold.

**Figure 3:**
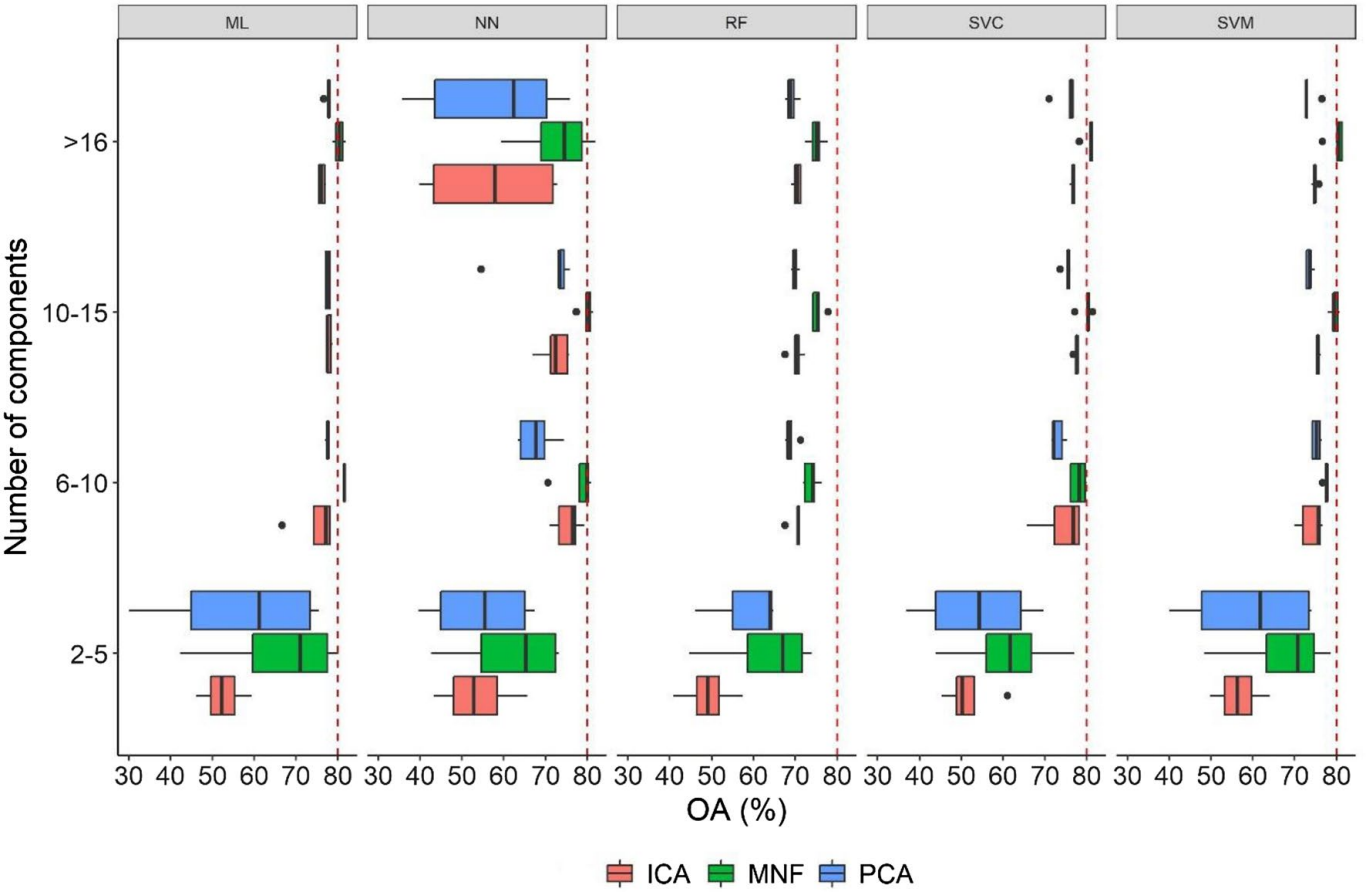
Overall accuracies (OA) of 300 models by the number of components, classification algorithms and dimension reduction types. Dashed line: 80% accuracy benchmark, *Ord* dimension reduction type, *MNF* Minimum Noise Fraction, *PCA* Principal Component Analysis, *ICA* Independent Component Analysis, *ML* Maximum Likelihood, *SVM* Support Vector Machine, *SVC* Linear type of SVM, *FR* Random Forest, *NN* Neural Net.

Multicriteria evaluation of the models revealed that the NN and RF classifiers were not efficient, but using > 10 components the SVC, the ML, and the SVM provided > 80% OAs with the MCs of the MNF transformation (Fig. 3). The ML classifier had the best model performance in absolute terms, and also regarding the one with the fewer components: 10 components, 83.3% OA. Regarding the classifiers and the DR types in the models having at least 80% OA, we found the following results: 45 models out of the 300 had > 80% OA; from this 45 model ML was found 19, SCV 11, NN 8 and SVM 7 times (as classification algorithms); furthermore, MNF was present 43, and PCA 2 times (as DR techniques).

### Post-classification with segmentation

**Figure 4:**
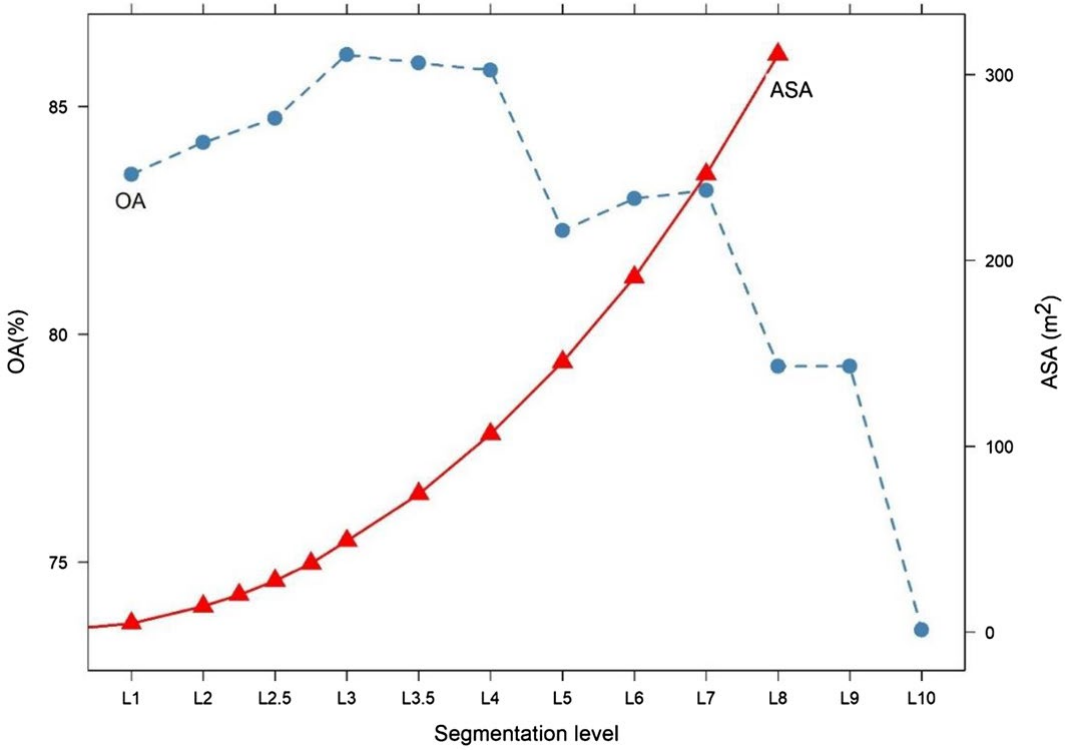
Relationship of segmentation levels, average segment areas (ASA), and overall accuracies (OA).

Post-classification was conducted in the most accurate classified image, which was the one produced with the ML classifier using 10 MCs as predictors (Fig. 4). Best result was gained with the L3 level (27.6 m^2^ average area and 86.1% OA), which was a 2.8% increase in the OA. At higher segmentation levels, L3.5 and L4 the decrease was minimal, but above L4 (50 m^2^ average segement size) there was a breakpoint in the OAs decreasing to 82.3% from 85.8%, and reached 73.5 at L10 (310 m^2^ average segment size).

**Figure 5:**
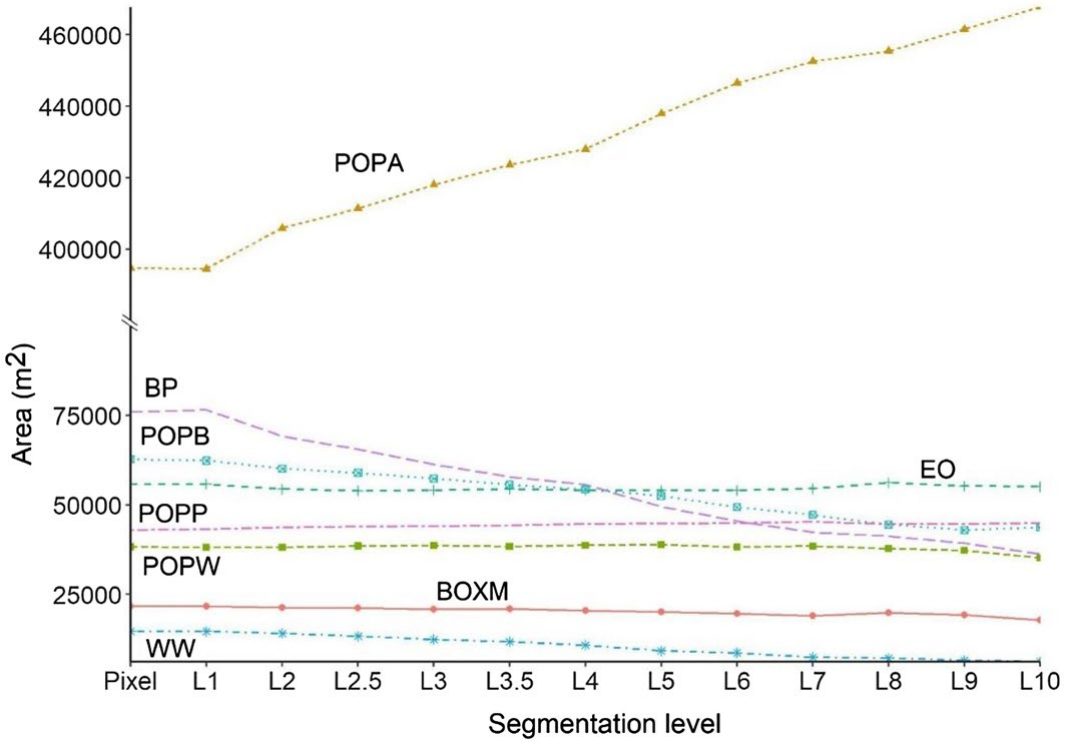
Area of tree species in the function of segmentation level. *WW* White willow, *POPA* Poplar (Agathe-F), *BP* Black pine, *POPW* White poplar, *BOXM* Boxelder maple, *POPP* Pannonia poplar, *POPB* Black poplar, *EO* English oak.

Analysis of tree species revealed that increasing segmentation level had different effect on the area change by classes: while POPA’s area increased by 18%, WW and BP decreased by 58 and 53%, POPB had 30%, and BOXM had 18% decrease, and the rest of the species’ (POPP, POPW and EO) are changed only ± 2–3% (Fig. 5). However, there was no direct relationship between the class level accuracy metrics (UA and PA) and the level of segmentation: class areas, UA and PA values had no relationship, correlation was 0.002 (*p* = 0.982). Generally, UAs were 15% larger than PAs on average, and the t-test indicated significant difference (t = 5.13, *p* < 0.001). In case of segmentation, differences were low and not significant (F = 0.29, *p* = 0.99).

**Figure 6:**
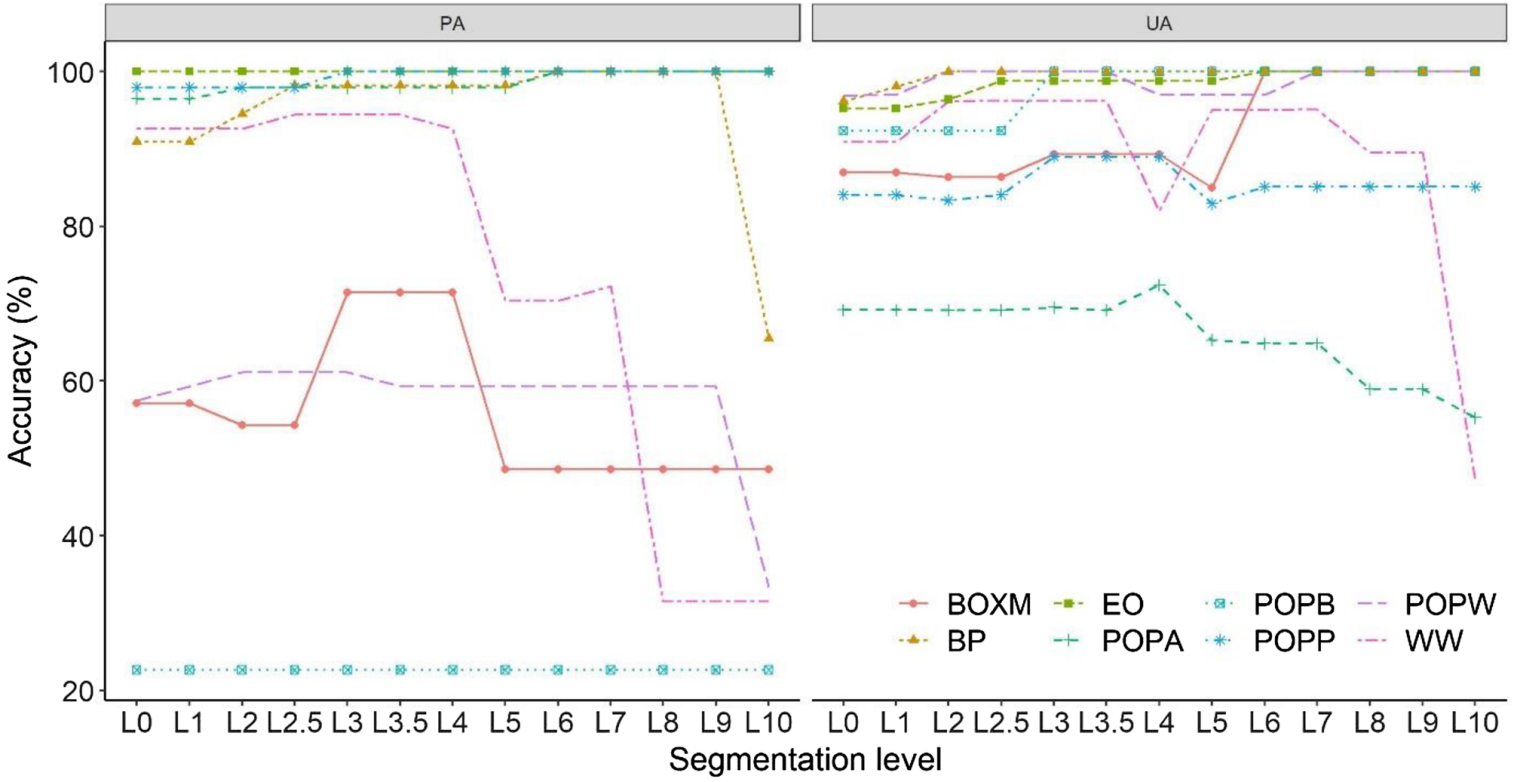
User’s accuracy (UA) and Producer’s Accuracy (PA) of tree species by segment levels. *WW* White willow, *POPA* Poplar (Agathe-F), *BP* Black pine, *POPW* White poplar, *BOXM* Boxelder maple, *POPP* Pannonia poplar, *POPB* Black poplar, *EO* English oak.

UA and PA relevantly differed by the segment levels and usually the change was decrease except in case of BOXM at PA values where it increased with 10.7%. Largest accuracies could be achieved at L3–L4, and similarly to OAs, most species had worse accuracy values with larger segment levels (Fig. 6). Despite the good spectral separability POPB–POPA and POPW–POPA showed the greatest mixting, which is also due to the fact that its occupied area has been increasing the most, thus overclassifying its own area. This also points out one of the problems with the MRS technique, as the area of the class occupying the largest area increases the most.

**Figure 7:**
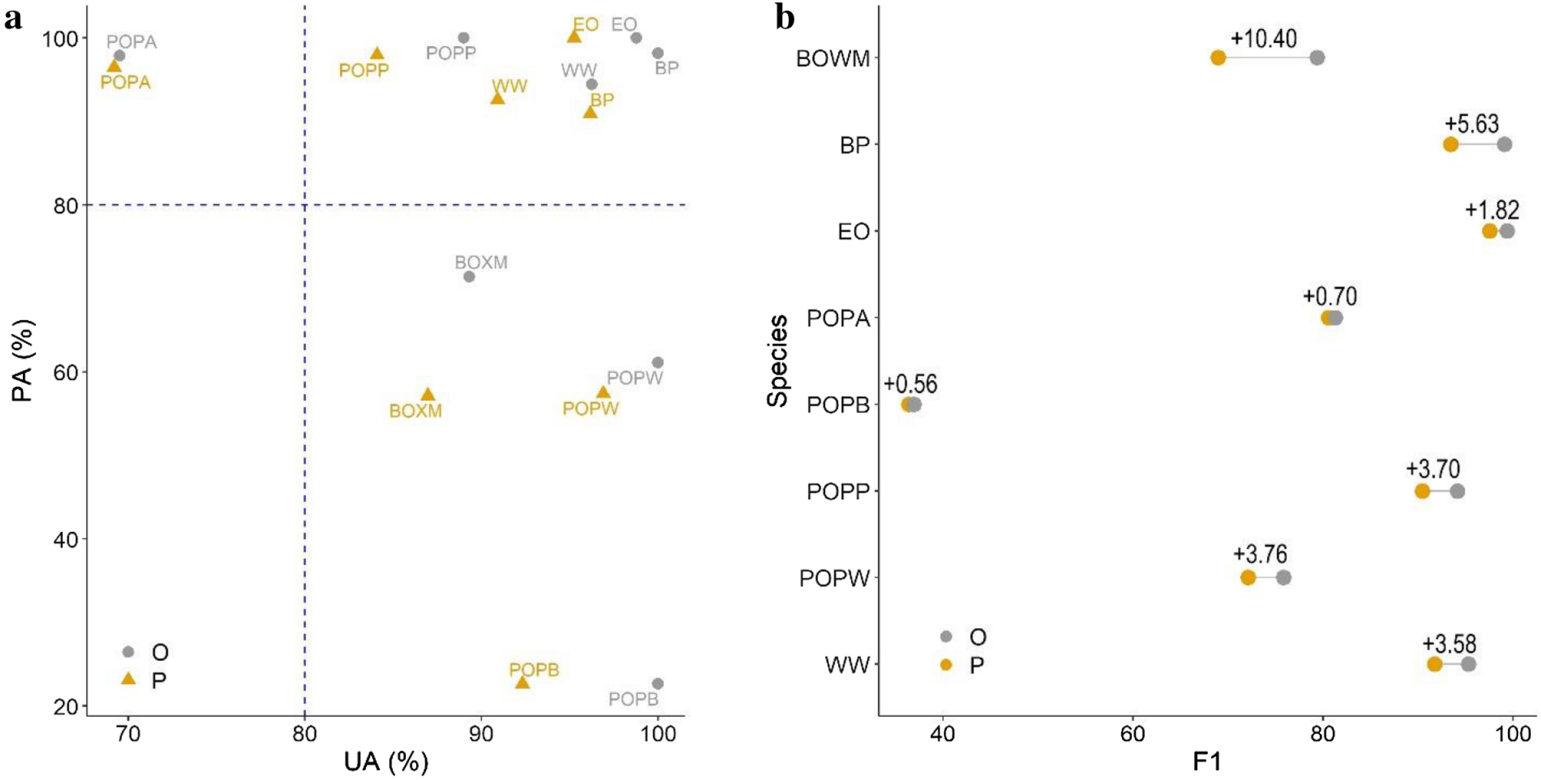
User’s accuracy (UA) and Producer’s accuracy (PA) of the species level classification (**a**) and F1-scores by species (**b**). 10 MCs and ML classifier and the post-classified image with L3 segments; *O* object-based post-classification, *P* pixel-based classification; *WW* White willow, *POPA* Poplar (Agathe-F), *BP* Black pine, *POPW* White poplar, *BOXM* Boxelder maple, *POPP* Pannonia poplar, *POPB* Black poplar, *EO* English oak; dashed line: 80% accuracy benchmark.

We compared the UA and PA values by tree species and found that WW, BP, EO, POP had PA above 90%, POPW and BOXM had 61–71%, and POPB had the lowest with 22%. UAs were usually larger, even the lowest value was 70% (POPA) and all other species were above 89% (Fig. 7a). POPB’s 100% UA and 22% PA indicated its possible overrepresention in the final map. None of the tree species had decreasing accuracy values after the object-based reclassification; however, it did not show increasing in all cases. F1-scores also showed increase after the post-classification at each species (Fig. 7b), and the difference was significant according to the Wilcoxon test (W = 36, z = 2.52, p_M-C_ = 0.007).

**Figure 8:**
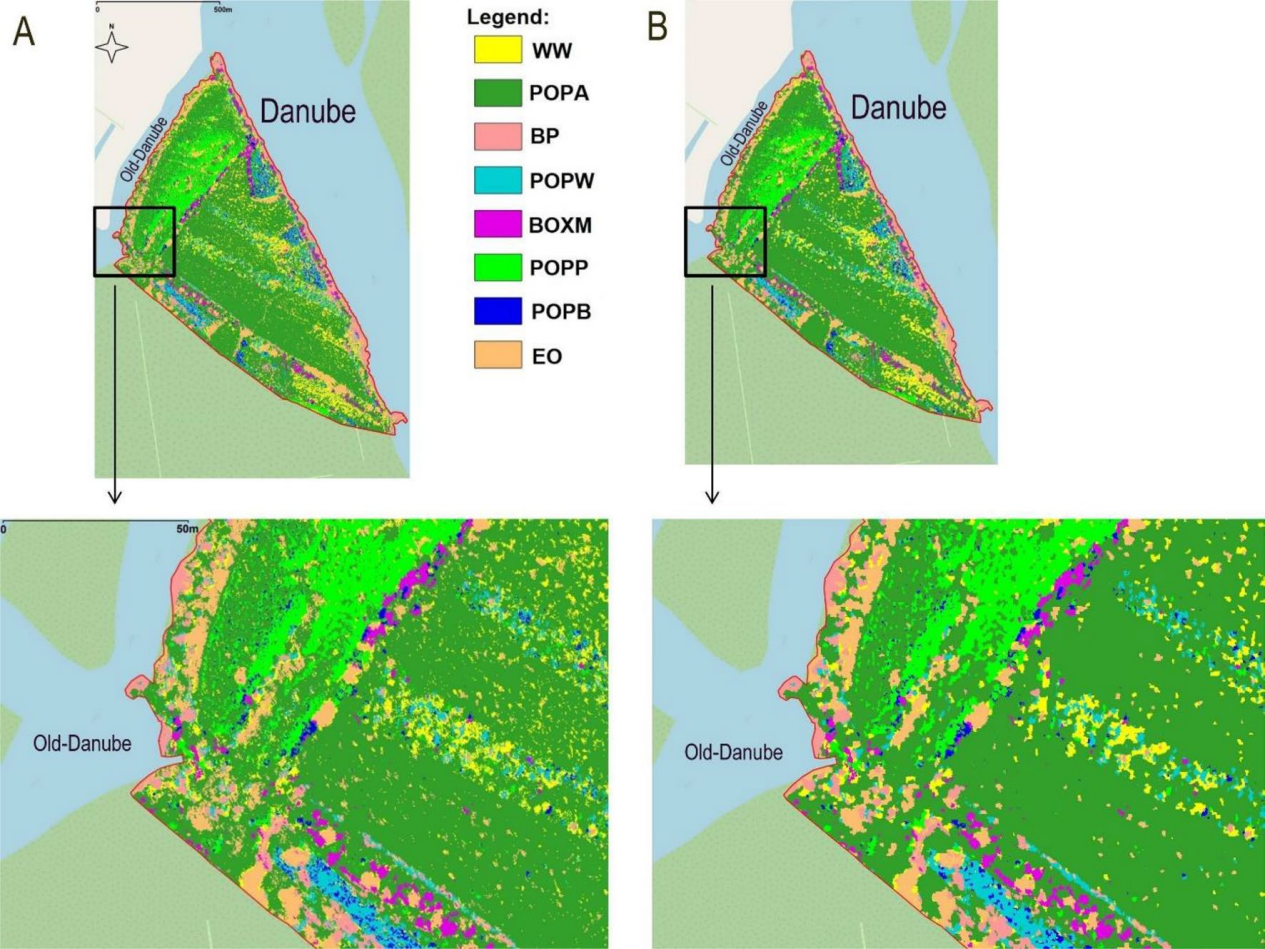
The comparison of pixel-based classification image (**A**) and object-based reclassified classification image (**B**). *WW* White willow, *POPA* Poplar (Agathe-F), *BP* Black pine, *POPW* White poplar, *BOXM* Boxelder maple, *POPP* Pannonia poplar, *POPB* Black poplar and *EO* English oak.

Pixel-based classification resulted in a map having several misclassified single pixels within tree crowns, which was almost eliminated during the post-classification (Fig. 8). Individual trees and tree groups were also better separated.

## Discussion

DR is an important preliminary step for hyperspectral image processing to handle the Hughes effect. Beside the most popular PCA and MNF, we involved the lesser-known ICA, as well. MNF performed better in the light of OAs than the other two techniques: median of the models had been run with MNF components was 78.2% OA, 72.6% with ICs and 72.8% with PCs. Hamada et al.^19^ and Priyadarshini et al.^22^ also found that MNF was the most efficient technique; however, Arslan et al.^23^ reported no significant difference among different DR methods, and Dabiri and Lang^21^ found ICA components better as input data. This latter research was similar to our current work (tree species classification with DR), but their method relied on super-pixel segmentation as a first step, while we used the segmentation (the MRS technique) only at the post-classification phase. Wang and Chang^18^ also came to the conclusion that ICA provides the best results, in their study they examined the same three different types of DR techniques. Ibarrola-Ulzurrun et al.^20^ also examined the three DR method, and their findings coincided with our result, MNF was the best, although, they examined land-cover classes, not specifically tree species with higher pixel value similarity among the classes. All DR techniques are based on the given dataset, and this seemingly contradicting results only highlights the varying efficiency of the methods depending on the data and target objects. Even the most popular and usually well performing techniques can be less effective than others under specific circumstances. In our case the MNF was the best, but it was the occasional consequence of the data charactersitics, which is, however, often occurs when it comes to hyperspectral data processing^24^. There are results on cases when PCA was not successful, and the DR was not reasonable technique to gain the best accuracies (e.g. Schlosser et al.^25^). Instead of using only one type of DR technique^8,15^, we revealed that there can be even 6% difference in the gained accuracies, thus, it may worth to compare the different ordinations as input data.

We found that ML outperformed the robust machine learning algorithms being considered more usable or efficient in other studies^26–29^. Our results justify that ML still has its role in image classification regardless the fact that machine learning and deep learning algortihms often outperforms it. If there are sufficient training pixels related to the number of bands, and the distributions are close to normal in the classes, ML can provide excellent outcomes. Accordingly, ML is not ideal with hyperspectral data, but DR is a good tool as a preliminary step to produce better inputs.

All classifiers were sensitive to the input number of bands (i.e. components), all algorithms performed differently. The NN and partly the RF were different from the others, because higher number of input bands resulted in lower OAs. The reason was that involving the higher number components caused bias in the trained model and were lesser useful, and NN and RF were the ones where optimization was crucial. However, usually both NN and RF provided the best OAs in the comparisons. We also revealed that there can be even 6% difference in the gained accuracies regarding the highest values depending on the number of the input components, so it may worth to compare the different type of classifiers depending on the study area.

Post-classification is not a new procedure in remote sensing, but there are no standard methods to perform it. While some authors suggested using textural information, spectral indices, ancillary data, visual interpretation, or smoothing^30–32^, we applied an object-based method, the MRS combined with a majority filter. It helped to overcome the misclassified pixels caused by the pixel-based classification (salt and pepper error), i.e. noise^33^. Segmentation parameters, especially the scale parameter relevantly affected the results, thus, testing of MRS is essential to achieve the highest accuracy, since the difference in OA values can be resulted in more than 10% among segmentation levels. Although post-classification did not result in outstandingly better accuracy (only 2–3%), but the class level accuracies were significantly better than the pixel-based method; furthermore, the procedure improved the quality and the readability of the final map.

The investigated area of the ‘Gemenc forest’ contains various plant species with a diverse flora which holds a rich fauna with a wide range of species and unique individuals^34,35^. Thus, classifications were tested in a diverse floodplain forest area; therefore, our results can be relevant not only for floodplain forest areas in the temperate zones, but the proposed approach (DR—classification—post-classification with comparative model selection) have also a great potential for any diverse forest conditions.

One of the major forest management aspects of our results can be associated the fact that several tree species, typical for temperate zone floodplain forests, were involved in this study. In the studied area, tree vegetation is determined by the river Danube: the characteristic trees belong to e.g. willow trees settled in the lower terrains, while e.g. poplar species in the higher terrains^36,37^. The selected tree species showed significant morphological and growth differences: e.g. willow is a hanging voluminous tree type, oaks grow slowly and is spacious, while poplar, maple and pine grow more quickly and higher^38,39^. In addition, the crown color intensity of these species also shows significant variability e.g. willow and poplar are lighter than the other species^38,39^. Under these conditions, the used classifiers combined with careful selection of DR and post-classification approaches showed 10% better OA than in previous studies^40,41^ providing a valuable practical option in the mapping of forest trees where there are populations of mixed tree species. Aerial imaging ensures tree composition maps with high accuracy and helps to monitor easily, quickly and more accurately the changes both spatially and temporally; especially in those areas were ground surveys can be difficult to conduct such as in floodplain forest areas^8,10,11^.

Of course, the approach provided in this study still needs improvements, but it shows a possible direction of more accurate data collections for understanding forest environmental conditions and for establishing more successful afforestation plans.

## Conclusions

Our aim was to propose an efficient novel strategy including the testing of DR technique, peformed on hyperspectral image, provides the best input data for tree-species classification algorithms, and if the post-classification was a useful step in gaining the final map. We found that MNF provided the best input data for the classifications, medians of OAs were 6% better than ICA and PCA, and this difference was significant. PCA’s and ICA’s components resulted in similar accuracies (72.2 and 72.8% OAs), which is also supported by the professional. 7–8 components were needed to get the highest OAs regarding all DR technique. ML classifier had the best OA combined with 8 MNF components (83.3). Finding the right combination of DR technique and classifier is important, it can cause more than a 10% change in the OAs, considering only the average differences. RF and NN were sensitive to higher number of components and their performance were uncertain having varying, and often lower OAs than SVC, SVM and ML. During post-classification, we determined the optimal segment size, gained by MRS, based on the OAs, and also found that class level metrics, i.e. UAs and PAs had the largest values with this area extent, too. We identified species can distinghuished with high accuracy (BP, EO, WW, POPP) and found that POPB is often misclassified with high commission errors. Post-classification improved the best OA with 3%; furthermore, eliminated most of the salt-and-pepper error of the pixel-based classification. We proved that traditional classifiers, such as ML, are able to gain high accuracies over the robust machine learning ones, the careful selection of DR, the number of components and a post-classification help to reach even 10% better accuracies. These results can be implemented in forest management or natue conservation helping them to uncover the structure and to maintain an economically and ecologically sustainable plantation or seminature forest.

## Materials and methods

### Study area

**Figure 9:**
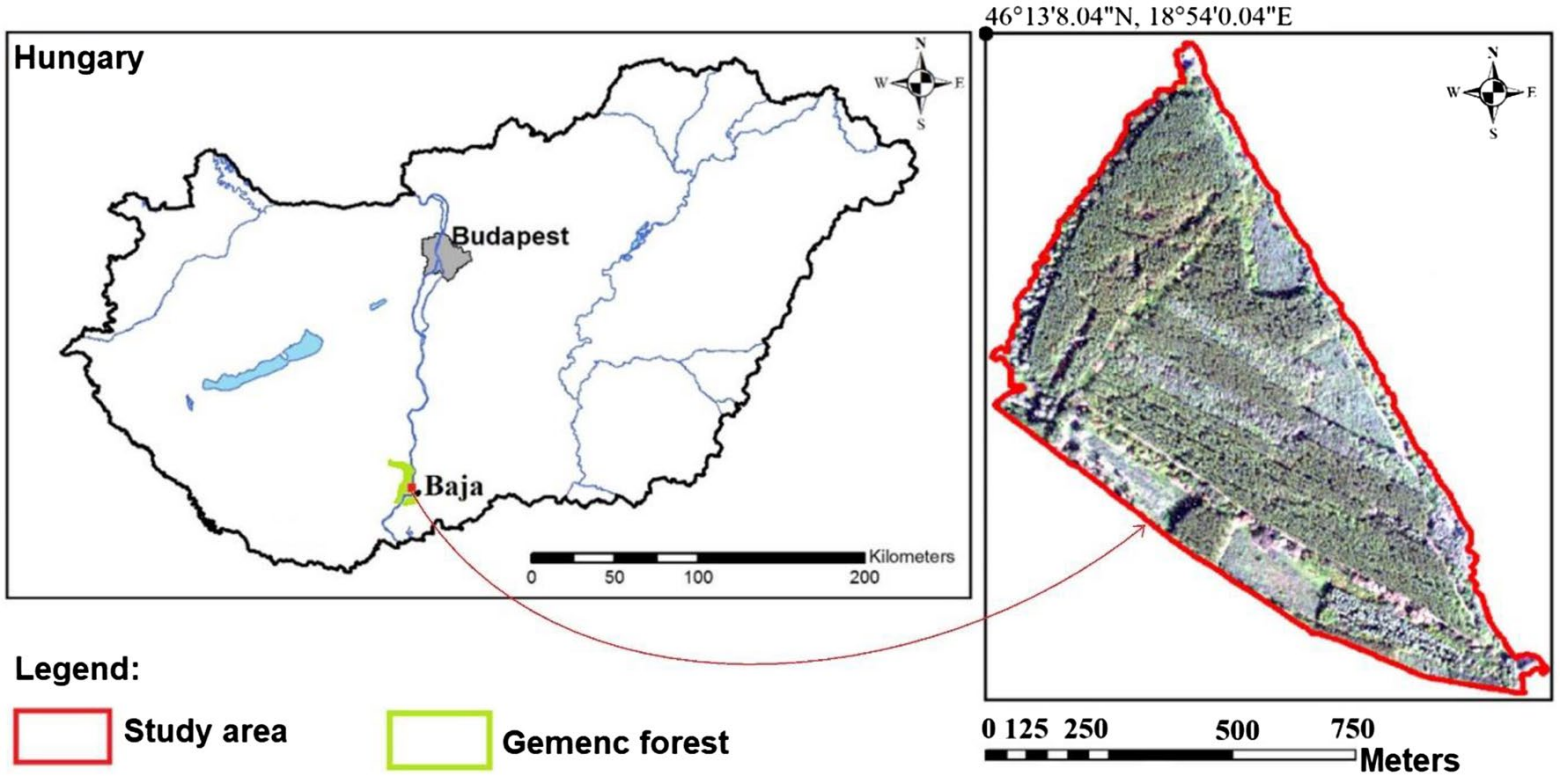
Location of the study area, the ‘Gemenc forest’, Hungary.

The study area, a part of the ’Gemenc forest’, was located in Hungary, near Baja city by the river Danube (46° 13′ 8.04″ N, 18° 54′ 0.04″ E) with the extent of 70 ha (Fig. 9). The area is a floodplain and was formed in the last 5000 years by the meandering Danube^36^. Danube is a primary factor in determining the flora and fauna along its course. During floods, 1–2 times a year, the river inundates the floodplain for weeks and forms a specific soil (Luvisol) and a habitat for plant and animal species of nature conservation. Regarding forestry, gallery forests with old natural trees and new plantations are the main elements of the land cover; thus, the favourable species distribution made possible the wood production and efficient forest management^36^.

Different tree species create various environments (e.g., sunlight conditions, nutrient and water availability) in the near-ground layer providing a wide range of flora and fauna of the given area (e.g. Nambiar and Sands^42^; Mayoral et al.^43^). This effect can be even more complex for micro and macro flora/fauna in floodplain forests^44^ such as in the investigated ‘Gemenc forest’ area having a various mixture of different trees (dominant tree species are summarized in Table 2) and plants and form a complex living community (e.g. Schöll et al.^34^; Ágoston-Szabó et al.^35^). Thus, knowing the compostion and distribution of trees, we also gain indirect information on the living communities and the species biodiversity (e.g. Vorster et al.^3^; Dyderski and Jagodzinski^45^; Dyderski and Pawlik^6^).

**Table 2:** Scientific name, common name and abbreviation of eight tree species (cultivars) assessed in the study area (Gemenc, Hungary, 2018).

Scientific name	Common name	Abbreviations
*Salix alba*	White willow	WW
*Populus* × *euramericana* cv. Agathe-F	Poplar (Agathe-F)	POPA
*Pinus nigra*	Black pine	BP
*Populus alba*	White poplar	POPW
*Acer negundo*	Boxelder maple	BOXM
*P. x euramericana 'Pannónia'*	Pannonia poplar	POPP
*Populus nigra*	Black poplar	POPB
*Quercus robur*	English oak	EO

### Hyperspectral data

The hyperspectral data were collected on 12 September, 2018, with an Asia Kestrel 10 sensor placed on a piloted aircraft, in the spectral range of 400–1000 nm in 178 spectral bands with a GSD of 1 m. Raw hyperspectral data were preprocessed with CaliGeoPRO for geometric and radiometric corrections. Noisy bands were removed based on visual selection. Majority of the noisy bands were at lower wavelength, thus, all bands < 470 nm were removed to avoid the bias in the statistical evaluation, which can reduce classification efficiancy^46^. The survey was a one-flight-lane; thus, the applied atmospheric correction method was the empirical line model, which provided an alternative to radiative transfer modelling approaches^47^.

### Reference dataset

Eight tree species were distinguished based on field observations and the visual analyzis of the aerial image where we checked that each observed tree crown was visible (Table 2). Reference data (1272 pixels) were recorded as polygons of tree individuals (areas covering the canopy of the identified individuals). Next, we randomly split the reference polygons into training and testing datasets in 60:40 ratios.

We used the Jeffries–Matusita (JM) distance to test the statistical spectral separability of the classes. JM distance is usually used in remote sensing and is similar to Bhattacharrya distance in the classical statistics; however, it enhances more the pairs of low separability^48^. JM distance values are close to 2 when signatures are completely different, and 0 indicates identical signatures. JM values indicated good separaibility.

### Image processing

**Figure 10:**
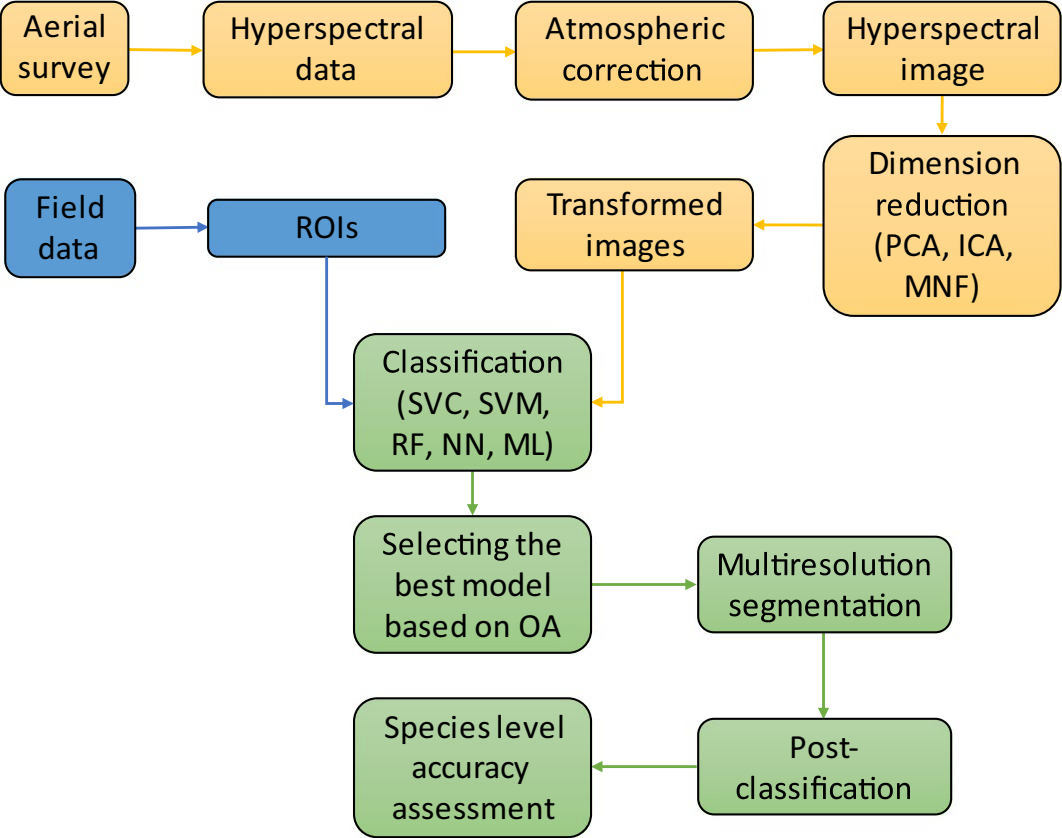
Workflow of image processing and post-classification. *ROIs* training pixels by Region of Interests, *OA* overall accuracy, *MNF* Minimum Noise Fraction, *PCA* Principal Component Analysis, *ICA* Independent Component Analysis, *ML* Maximum Likelihood, *SVM* Support Vector Machine, *SVC* Linear type of SVM, *RF* Random Forest, *NN* Neural Net.

Image processing had three steps: feuture extraction, classification with machine learning algorithms and post-classification (Fig. 10).

### Dimension reduction

Basic concept of all ordinations are to create a new feature space with artificial variables (i.e. components) keeping the maximum explained variance. One of the most popular DR techniques is the Principal Component Analysis (PCA), which projects the large number of variables (such as bands of hyperspectral images) into orthogonal principal components (PCs; non-correlating variables)^26,49^. We emphesize that not all datasets are suitable to perform a successful PCA, due to assumptions of normal distribution, linearity, and variables have to be correlated, hyperspectral images usually meet the preconditions. Number of PCs equal to the number of variables (i.e., bands), but the useful information is concentrated in the first 10–20 PCs. Eigenvectors define the multidimensional space, and eigenvalues reflect their relevance: according to the Kaiser’s rule^50^, PCs of eigenvalues > 1 were considered as input data. Number of useful PCs differs by the studied areas, and the images.

An extension of PCA, the Independent Component Analysis (ICA) decorrelates the signals (2nd order statistics) and reduces higher-order statistical dependencies, and finally transforms the original variables to independent components (ICs)^51–53^. ICA transformation does not require normal distribution for the variables. The main assumption is that of variables can be decomposed into non-Gaussian and statistically independent subgroups. While PCA compresses the information providing uncorrelated components (PCs), ICA separates the ordination space into independent components (ICs)^54^.

In remote sensing, Minimum Noise Fraction (MNF) transformation is the most popular technique for DR^13,22^. The MNF transformation consists of two PCAs, the first estimates the spectral noise using the covariance matrix, and the second rotation uses the decorrelated and rescaled components, and gives priority to the higher, eigenvalues, which have large explained variance^19^. The outcome is a set of MNF-components (MCs). MNF is widely used because usually provides the best input data for classifications^19^.

### Classification algorithms

We investigated the efficiency of five classification algorithms: Maximum Likelihood (ML), Random Forest (RF), Neural Net (NN), Support Vector Machine (SVM) with Radial Basis Function (RBF), and the linear Support Vector Classifier (SVC).

The ML algorithm uses the standard deviation and the covariance matrix for each specific class during classification to calculate the chance of the data falling into those groups^27^. However, the ’Hughes phenomenon’ limits the application of this classifier with hyperspectral data when the training dataset is small related to high number of spectral bands^12,27^. Thus, dimensional reduction was an essential step, given that the training datasets were not sufficent to run the classifier on the original 178 bands of the image. Due to the use of the variance–covariance matrix within the class distributions, it can achieve better results on normally distributed data than other parametrized classifications^55^. Scale factor was set to 1.

SVC and SVM classifiers use a multidimensional “hyperplane” to separate the pixels of the data and create the classes, the hyperplane is positioned to maximize the distance between the nearest training data and the separation plane^56^. Since the separation planes are linear in all cases, the non-linear boundaries cause errors in the classification (the SVC uses this linear planes), and in several cases the border cannot be delineated with a linear plane, the SVM model uses the “kernel-trick” parameterization to overcome the issue^56^. We applied two types of parameterizations: (i) SVM with the radial basis function (SVM), and (ii) SVM with linear regularization parameter (SVC). Recently, the SVM classification had become popular, as it often provides outstanding classification accuracy^28^. Hyperparameters are the gamma (for RBF kernel) and C (penalty parameter) and was determined by grid search. Gamma was chosen using a search between 0.05 and 0.5 based on the inverse of the number of involved bands, and the C was set to 100 after tested on a logarithmic scale of 0.001–1000 with the increment of magnitude (0.001, 0.01. 0.1, 1, 10, 100 and 1000). The SVC was conducted with the same testing of the C parameter.

RF is a non-parametric classification algorithm based on decision trees; each tree is built on a random partion of data taken from the reference dataset with bootstrapping. 36.7% of the training data is left out of this process, randomly by decision trees, which is kept to calculate the out-of-bag (OOB) error: the model also classifies the OOB data and evaluates the overall accuracy. Number of decision trees and variables can be defined by the user, whereas we applied 10 decision trees and also calculated the OOBs. RF’s performance, similarly to ML, is sensitive to small cases in the training dataset^29^.

The design of Artificial Neural Networks (NN) uses non-linear processing units; i.e. neurons. Neurons have three layers: at least one input, at least one hidden, and an output layer. Between the neurons there is a weight-derived network, which complexity depends on the applied algorithm and input data structure^57^. We applied a nonlinear layer feed-forward model with standard dissemination for supervised learning with 1 hidden layer and 10,000 training iterations. Furthermore, the training threshold contribution was 0.9, the training rate 0.2, and training momentum was 0.9 with 0.1 RMS exit criteria^24^.

Each classifier had been run with the 2–20 components of the three DR techniques (PCA, ICA, and MNF) in order to find the most accurate tree species map. DRs and classifications were conducted in the Exelis Envi 5.1 and QGIS 3.6 with EnMAP-Box 3 extension (www.l3harrisgeospatial.com, www.qgis.org, plugins.qgis.org/plugins/enmapboxplugin/).

### Post-classification

Object-based Image Analysis (OBIA), i.e. segmentation, is an important alternative over the pixel-based solutions in vegetation mapping research^58^. Images are divided into small homogenous regions (segments) based on the pixel-homogeneity in one or more dimensions^59^. One of the most efficient procedure is the multiresolution segmentation (MRS): using the scale, shape, and compactness parameters, the algorithm determines segments from the pixels in an iterative process. Many studies used the MRS on forest areas as the basis of OBIA^15,60,61^.

We applied the MRS technique on the classified image (Fig. 2). The scale parameter was tested from 1 to 10 (L1–L10 Levels) to find highest overall accuracy (OA). Visual interpretation was applied to select the optimal shape and compactness parameters (i.e. finding the suitable shapes for tree crowns), and finally we found the 0.5 compactness and 0.3 shape values the most reasonable parameters. We then reclassified the image with the majority pixel value of the inner area of each object to filter out the misclassified pixels. Reclassification was carried out on the pixel-based classified image using a 16-bit greyscale image, each class got its value from 1 to 8 (i.e., the codes of the tree species); thus, segmentation succesfully built the objects, i.e. integrated the strikingly different pixels. The reclassification aimed to eliminate the obviously misclassified pixels in each segment; the most frequent value of the class that covered the largest area within the object was extended to each segment.

### Accuracy assessment

Classifications were evaluated with thematic accuracy indicators to examine the results of our results after classifications: overall accuracy (OA), user’s accuracy (UA), producer’s accuracy (PA), and the errors of commission and omission^62,63^. F1-scores were also calculated as a harmonic mean of UA and PA^64^.

### Statistical evaluation of the classification results

Accuracy measures were evaluated with statistical tests. Normal distribution of the dependent variable (OA, UA, PA) was checked with the Shapiro–Wilk test. We applied General Linear Model (GLM), in this case a 3-way ANOVA, to reveal the importance of DR techniques, number of components and the classifiers (H0: means had no difference in the seven combinations of the three factors, H1: means were not equal). Model parameters, and the effect sizes were reported, the latter expressed as partial ω^2^p, which indicated the contribution of the variables and the interaction of the factorial variables as a standardized metric^65^. Effect can be very small (ω^2^p < 0.01), small (0.01 > ω^2^p > 0.06), medium (0.06 > ω^2^p > 0.14), and large (ω^2^p > 0.14). We also applied t-test and ANOVA using 9999 Monte-Carlo permutation using class level accuracies as dependent variables, and the type of accuracy metric (User’s Accuracy, Producer’s Accuracy) and the level of segmentation were the independent variables, respectively. Pearson correlation was used to analyze the relationship between the average segment areas and overall accuracies in the post-classification. Change in F1-scores was studied by the Wilcoxon test with Monte-Carlo permutation (n = 9999); H0 was that medians of F1-values were equal both for the pixel-based and the post-classified approaches. Statistical analysis had been conducted with R 4.1^66^ with the gamlj package^67^.

## Supplementary Information

Supplementary Information.

## Data Availability

The datasets used during the current study available from the corresponding author on reasonable request.
