# Supplementary material for: Tree species composition mapping with dimension reduction and post-classification using very high-resolution hyperspectral imaging
Source: Sci Rep. 2022 Dec 3;12:20919. doi: 10.1038/s41598-022-25404-x (PMC9719473; doi:10.1038/s41598-022-25404-x)
Supplement: Supplementary file 1 — Supplementary Information. [file 41598_2022_25404_MOESM1_ESM.pdf]

**Supplementary table 1**. Samples of previous studies on tree species classification, dimension reduction and post-classification using hyperspectral imaging related to forest tree species.

| Topic | Year | Publication sample |
|---|---|---|
| Tree species identification using hyperspectral imaginary and or UAV based technology and/or LiDAR data | 2007 | Hamada et al.[19] |
| | 2010 | Jones et al.[40] |
| | 2012 | Latifi et al.[26] |
| | 2014 | Dalponte et al.[60] |
| | 2016 | Fassnacht et al.[13] |
| | 2016 | Richter et al.[11] |
| | 2018 | Dabiri & Lang[21] |
| | 2019 | Burai et al.[15] |
| | 2019 | Sothe et al.[41] |
| | 2020 | Modzelewska et al.[8] |
| | 2020 | Zhao et al.[58] |
| | 2021 | Vangi et al.[14] |
| | 2021 | Li et al.[17] |
| Dimension reduction with hyperspectral image analysis | 2006 | Wang & Chang[18] |
| | 2017 | Ibarrola-Ulzurrun et al.[20] |
| | 2019 | Priyadarshini et al.[22] |
| | 2020 | Arslan et al.[23] |
| Improving the accuracy using post-classification enhancement | 2009 | Manandhar et al.[30] |
| | 2017 | Thakkar et al.[31] |
| | 2016 | El-Hattab[32] |
